# *N*,*N*′-Diethyl-*N*,*N*′-[1,3-phenylene­bis(methyl­ene)]dibenzene­sulfonamide

**DOI:** 10.1107/S1600536811040700

**Published:** 2011-10-29

**Authors:** Ejaz, Islam Ullah Khan, Hira Ahmad, William T. A. Harrison, Tahir Ali Sheikh

**Affiliations:** a Materials Chemistry Laboratry, Department of Chemistry, GC University, Lahore 54000, Pakistan; b Department of Chemistry, University of Aberdeen, Meston Walk, Aberdeen AB24 3UE, Scotland; c Materials Chemistry Laboratry, Department of Chemistry, GC University, Lahore-54000, Pakistan

## Abstract

In the title compound, C_24_H_28_N_2_O_4_S_2_, the dihedral angles between the central benzene ring and the pendant rings are 77.44 (11) and 79.23 (10)°, and the dihedral angle between the pendant rings is 23.31 (12)°. Both sulfonamide groups project to the same side of the central benzene ring and the mol­ecule has approximate non-crystallographic mirror symmetry. One of the ethyl side chains is disordered over two sets of sites in a 0.526 (14):0.474 (14) ratio. In the crystal, inversion dimers linked by pairs of weak C—H⋯O inter­actions occur, generating *R*_2_^2^(28) loops.

## Related literature

For a related structure, see: Khan *et al.* (2011 ▶).
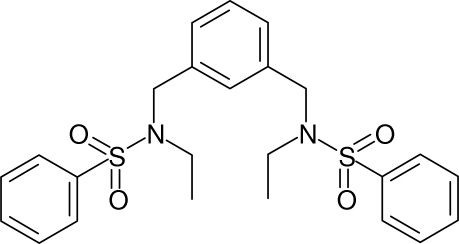


## Experimental

### 

#### Crystal data

C_24_H_28_N_2_O_4_S_2_
*M* *_r_* = 472.60
Monoclinic, 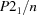

*a* = 9.1865 (3) Å
*b* = 19.0679 (7) Å
*c* = 14.3870 (5) Å
β = 106.122 (1)°
*V* = 2421.02 (15) Å^3^
*Z* = 4
Mo *K*α radiation
μ = 0.25 mm^−1^
*T* = 296 K
0.13 × 0.10 × 0.09 mm

#### Data collection

Bruker APEXII CCD diffractometer
23272 measured reflections
6013 independent reflections
4188 reflections with *I* > 2σ(*I*)
*R*_int_ = 0.025

#### Refinement

*R*[*F* ^2^ > 2σ(*F* ^2^)] = 0.045
*wR*(*F* ^2^) = 0.132
*S* = 1.01
6013 reflections
290 parameters
H-atom parameters constrained
Δρ_max_ = 0.29 e Å^−3^
Δρ_min_ = −0.38 e Å^−3^

### 

Data collection: *APEX2* (Bruker, 2007 ▶); cell refinement: *SAINT* (Bruker, 2007 ▶); data reduction: *SAINT* (Bruker, 2007 ▶); program(s) used to solve structure: *SHELXS97* (Sheldrick, 2008 ▶); program(s) used to refine structure: *SHELXL97* (Sheldrick, 2008 ▶); molecular graphics: *ORTEP-3* (Farrugia, 1997 ▶); software used to prepare material for publication: *SHELXL97* (Sheldrick, 2008 ▶).

## Supplementary Material

Crystal structure: contains datablock(s) I, global. DOI: 10.1107/S1600536811040700/su2324sup1.cif

Structure factors: contains datablock(s) I. DOI: 10.1107/S1600536811040700/su2324Isup2.hkl

Supplementary material file. DOI: 10.1107/S1600536811040700/su2324Isup3.cml

Additional supplementary materials:  crystallographic information; 3D view; checkCIF report

## Figures and Tables

**Table 1:** Hydrogen-bond geometry (Å, °)

*D*—H⋯*A*	*D*—H	H⋯*A*	*D*⋯*A*	*D*—H⋯*A*
C17—H17⋯O1^i^	0.93	2.57	3.409 (3)	151

Symmetry code: (i) 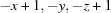.

**Fig. 1.**
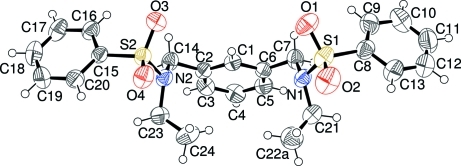
The molecular structure of the title molecule, showing the numbering scheme and 50% displacement ellipsoids. Only one orientation of the disordered atom C22 and its attached H atoms is shown.

**Fig. 2.**
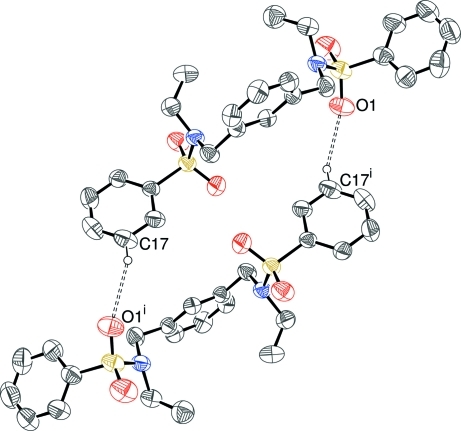
A view of the inversion dimer in the crystal structure of the title compound. All H atoms except H17 have been omitted for clarity, and the C—H···O interactions are shown as dashed lines [Symmetry code: (i) 1–x, –y, 1–z].

## supplementary crystallographic information

### Comment

As part of our ongoing structural studies of sulfonamides (Khan *et al.*, 2011), the synthesis and structure of the title compound, (I), (Fig. 1), are now described.

The dihedral angles between the central benzene ring and the pendant rings are almost equal at 77.44 (11) and 79.23 (10)° and the dihedral angle between the pendant rings is 23.31 (12)°. Both sulfonamode groups project to the same side of the central ring and the molecule has approximate non-crystallographic mirror symmetry. The C8—S1—N1—C7 and C15—S2—N2—C14 torsion angles are -66.87 (15) and 70.98 (15)°, respectively. The S1—N1—C7—C6 and S2—N2—C14—C2 trsion angles are -148.14 (14) and 144.01 (14)°, respectively. One of the terminal methyl groups is disordered over two sets of sites in a 0.526 (14):0.474 (14) ratio.

In the crystal, inversion dimers linked by pairs of weak C—H···O interactions occur (Fig. 2, Table 1).

### Experimental

A mixture of *N*,*N*'-diethyl-(benzene-1,3-diyldimethanediyl)dibenzenesulfonamide (0.2 g, 0.43 mmol), sodium hydride (0.21 g; 0.88 mmol) and N, *N*-dimethylformamide (10.0 ml) was stirred in a 100-ml RB flask at room temperature for half an hour followed by the addition of ethyl iodide (0.134 g; 0.86 mmol). The reaction mixture was further stirred for five hours, and its completion was monitored by TLC. After completion, the contents were poured over crushed ice. The precipitated product was isolated, washed and crystallized from methanol to yield colourless blocks of (I).

### Refinement

The N-bound H atom was located in a difference Fourier map and its position was freely refined with the constraint *U*_iso_(H) = 1.2*U*_eq_(N). The C-bound hydrogen atoms were placed in calculated positions (C—H = 0.93–0.97 Å) and refined as riding atoms with *U*_iso_(H) = 1.2*U*_eq_(C). One of the ethyl side chains is disordered over two sets of sites in a 0.526 (14):0.474 (14) ratio for atoms C22A:C22B, which were refined isotropically.

### Crystal data

**Table:**

C_24_H_28_N_2_O_4_S_2_	*F*(000) = 1000
*M**_r_* = 472.60	*D*_x_ = 1.297 Mg m^−^^3^
Monoclinic, *P*2_1_/*n*	Mo *K*α radiation, λ = 0.71073 Å
Hall symbol: -P 2yn	Cell parameters from 6013 reflections
*a* = 9.1865 (3) Å	θ = 2.6–28.3°
*b* = 19.0679 (7) Å	µ = 0.25 mm^−^^1^
*c* = 14.3870 (5) Å	*T* = 296 K
β = 106.122 (1)°	Block, colourless
*V* = 2421.02 (15) Å^3^	0.13 × 0.10 × 0.09 mm
*Z* = 4	

### Data collection

**Table:**

Bruker APEXII CCD diffractometer	4188 reflections with *I* > 2σ(*I*)
Radiation source: fine-focus sealed tube	*R*_int_ = 0.025
graphite	θ_max_ = 28.3°, θ_min_ = 2.6°
ω scans	*h* = −12→8
23272 measured reflections	*k* = −24→25
6013 independent reflections	*l* = −17→19

### Refinement

**Table:**

Refinement on *F*^2^	Primary atom site location: structure-invariant direct methods
Least-squares matrix: full	Secondary atom site location: difference Fourier map
*R*[*F*^2^ > 2σ(*F*^2^)] = 0.045	Hydrogen site location: inferred from neighbouring sites
*wR*(*F*^2^) = 0.132	H-atom parameters constrained
*S* = 1.01	*w* = 1/[σ^2^(*F*_o_^2^) + (0.060*P*)^2^ + 0.7693*P*] where *P* = (*F*_o_^2^ + 2*F*_c_^2^)/3
6013 reflections	(Δ/σ)_max_ < 0.001
290 parameters	Δρ_max_ = 0.29 e Å^−^^3^
0 restraints	Δρ_min_ = −0.38 e Å^−^^3^

### Special details

**Table:**

Geometry. All e.s.d.'s (except the e.s.d. in the dihedral angle between two l.s. planes) are estimated using the full covariance matrix. The cell e.s.d.'s are taken into account individually in the estimation of e.s.d.'s in distances, angles and torsion angles; correlations between e.s.d.'s in cell parameters are only used when they are defined by crystal symmetry. An approximate (isotropic) treatment of cell e.s.d.'s is used for estimating e.s.d.'s involving l.s. planes.
Refinement. Refinement of *F*^2^ against ALL reflections. The weighted *R*-factor *wR* and goodness of fit *S* are based on *F*^2^, conventional *R*-factors *R* are based on *F*, with *F* set to zero for negative *F*^2^. The threshold expression of *F*^2^ > σ(*F*^2^) is used only for calculating *R*-factors(gt) *etc*. and is not relevant to the choice of reflections for refinement. *R*-factors based on *F*^2^ are statistically about twice as large as those based on *F*, and *R*- factors based on ALL data will be even larger.

### Fractional atomic coordinates and isotropic or equivalent isotropic displacement parameters (Å^2^)

**Table:**

	*x*	*y*	*z*	*U*_iso_*/*U*_eq_	Occ. (<1)
C1	0.78689 (19)	0.13374 (9)	0.68904 (13)	0.0448 (4)	
H1	0.7268	0.1480	0.6288	0.054*	
C2	0.73697 (19)	0.08014 (9)	0.73745 (14)	0.0449 (4)	
C3	0.8266 (2)	0.05935 (11)	0.82682 (15)	0.0554 (5)	
H3	0.7942	0.0233	0.8598	0.066*	
C4	0.9639 (2)	0.09163 (13)	0.86768 (15)	0.0614 (5)	
H4	1.0237	0.0775	0.9281	0.074*	
C5	1.0123 (2)	0.14489 (11)	0.81882 (15)	0.0545 (5)	
H5	1.1049	0.1666	0.8466	0.065*	
C6	0.92480 (19)	0.16639 (9)	0.72889 (14)	0.0452 (4)	
C7	0.9798 (2)	0.22228 (10)	0.67292 (17)	0.0557 (5)	
H7A	1.0849	0.2329	0.7054	0.067*	
H7B	0.9751	0.2045	0.6090	0.067*	
C8	1.0539 (3)	0.36406 (11)	0.56934 (15)	0.0582 (5)	
C9	1.1599 (3)	0.32027 (13)	0.54866 (18)	0.0702 (6)	
H9	1.1345	0.2745	0.5280	0.084*	
C10	1.3050 (3)	0.34546 (17)	0.5591 (2)	0.0881 (8)	
H10	1.3785	0.3162	0.5467	0.106*	
C11	1.3404 (4)	0.41342 (19)	0.5877 (2)	0.0957 (10)	
H11	1.4381	0.4299	0.5947	0.115*	
C12	1.2346 (4)	0.45677 (18)	0.6058 (2)	0.1002 (10)	
H12	1.2595	0.5030	0.6238	0.120*	
C13	1.0910 (3)	0.43270 (13)	0.5975 (2)	0.0796 (7)	
H13	1.0188	0.4623	0.6107	0.096*	
C14	0.5875 (2)	0.04462 (10)	0.69110 (16)	0.0517 (5)	
H14A	0.5908	0.0235	0.6304	0.062*	
H14B	0.5708	0.0076	0.7332	0.062*	
C15	0.22235 (19)	0.00993 (9)	0.59280 (13)	0.0437 (4)	
C16	0.2729 (2)	−0.05564 (10)	0.57526 (16)	0.0553 (5)	
H16	0.3584	−0.0602	0.5531	0.066*	
C17	0.1955 (3)	−0.11417 (11)	0.59097 (18)	0.0676 (6)	
H17	0.2282	−0.1586	0.5790	0.081*	
C18	0.0694 (3)	−0.10715 (13)	0.62447 (19)	0.0728 (7)	
H18	0.0180	−0.1469	0.6356	0.087*	
C19	0.0198 (3)	−0.04250 (14)	0.64141 (18)	0.0697 (6)	
H19	−0.0661	−0.0383	0.6633	0.084*	
C20	0.0957 (2)	0.01662 (11)	0.62643 (16)	0.0558 (5)	
H20	0.0623	0.0608	0.6388	0.067*	
C21	0.9119 (3)	0.32830 (13)	0.75365 (18)	0.0700 (6)	
H21A	0.9693	0.3704	0.7501	0.084*	
H21B	0.9692	0.3008	0.8083	0.084*	
C22A	0.7607 (7)	0.3480 (4)	0.7682 (5)	0.079 (2)*	0.526 (14)
H22A	0.7765	0.3746	0.8269	0.119*	0.526 (14)
H22B	0.7046	0.3063	0.7725	0.119*	0.526 (14)
H22C	0.7048	0.3759	0.7145	0.119*	0.526 (14)
C22B	0.7963 (9)	0.3175 (6)	0.8056 (7)	0.095 (3)*	0.474 (14)
H22D	0.8201	0.3458	0.8630	0.143*	0.474 (14)
H22E	0.7947	0.2690	0.8231	0.143*	0.474 (14)
H22F	0.6987	0.3306	0.7645	0.143*	0.474 (14)
C23	0.4252 (2)	0.12225 (12)	0.76072 (16)	0.0597 (5)	
H23A	0.3194	0.1129	0.7553	0.072*	
H23B	0.4857	0.0974	0.8171	0.072*	
C24	0.4538 (4)	0.19853 (14)	0.7750 (2)	0.0879 (8)	
H24A	0.5597	0.2078	0.7849	0.132*	
H24B	0.4240	0.2138	0.8307	0.132*	
H24C	0.3961	0.2234	0.7189	0.132*	
S1	0.87268 (6)	0.33183 (3)	0.56484 (4)	0.05927 (16)	
S2	0.32295 (5)	0.08483 (2)	0.57589 (4)	0.04724 (14)	
N1	0.88989 (17)	0.28716 (8)	0.66325 (12)	0.0512 (4)	
N2	0.46183 (16)	0.09500 (8)	0.67317 (12)	0.0484 (4)	
O1	0.82899 (19)	0.28392 (9)	0.48611 (12)	0.0765 (5)	
O2	0.77734 (19)	0.39036 (9)	0.56817 (15)	0.0850 (5)	
O3	0.38878 (17)	0.07049 (8)	0.49896 (11)	0.0615 (4)	
O4	0.22630 (16)	0.14401 (7)	0.57007 (12)	0.0634 (4)	

### Atomic displacement parameters (Å^2^)

**Table:**

	*U*^11^	*U*^22^	*U*^33^	*U*^12^	*U*^13^	*U*^23^
C1	0.0399 (8)	0.0470 (10)	0.0441 (10)	0.0051 (7)	0.0063 (7)	−0.0007 (8)
C2	0.0399 (8)	0.0434 (10)	0.0506 (10)	0.0043 (7)	0.0113 (8)	−0.0030 (8)
C3	0.0532 (11)	0.0572 (12)	0.0570 (12)	0.0080 (9)	0.0173 (9)	0.0120 (9)
C4	0.0490 (10)	0.0816 (15)	0.0479 (11)	0.0100 (10)	0.0041 (9)	0.0077 (10)
C5	0.0358 (8)	0.0674 (13)	0.0560 (12)	0.0034 (8)	0.0057 (8)	−0.0092 (10)
C6	0.0385 (8)	0.0441 (10)	0.0540 (10)	0.0041 (7)	0.0145 (8)	−0.0031 (8)
C7	0.0427 (9)	0.0502 (11)	0.0763 (14)	0.0030 (8)	0.0200 (9)	0.0027 (10)
C8	0.0683 (12)	0.0491 (11)	0.0539 (12)	−0.0051 (9)	0.0117 (10)	0.0015 (9)
C9	0.0817 (16)	0.0574 (13)	0.0753 (15)	−0.0027 (11)	0.0281 (13)	0.0091 (11)
C10	0.0849 (18)	0.096 (2)	0.094 (2)	0.0008 (16)	0.0428 (16)	0.0184 (16)
C11	0.091 (2)	0.110 (3)	0.089 (2)	−0.0403 (19)	0.0303 (17)	0.0000 (18)
C12	0.119 (2)	0.087 (2)	0.106 (2)	−0.0455 (19)	0.049 (2)	−0.0221 (17)
C13	0.0994 (19)	0.0578 (14)	0.0856 (18)	−0.0164 (13)	0.0322 (15)	−0.0105 (13)
C14	0.0459 (9)	0.0407 (10)	0.0667 (12)	0.0010 (8)	0.0123 (9)	−0.0021 (9)
C15	0.0380 (8)	0.0434 (9)	0.0468 (10)	−0.0011 (7)	0.0072 (7)	−0.0072 (8)
C16	0.0461 (10)	0.0473 (11)	0.0698 (13)	0.0003 (8)	0.0116 (9)	−0.0116 (9)
C17	0.0636 (13)	0.0445 (11)	0.0858 (17)	−0.0032 (10)	0.0061 (12)	−0.0072 (11)
C18	0.0654 (14)	0.0633 (14)	0.0829 (17)	−0.0207 (11)	0.0091 (12)	0.0073 (12)
C19	0.0542 (12)	0.0799 (17)	0.0783 (16)	−0.0141 (11)	0.0240 (11)	−0.0028 (13)
C20	0.0475 (10)	0.0573 (12)	0.0639 (13)	−0.0006 (9)	0.0174 (9)	−0.0110 (10)
C21	0.0774 (15)	0.0623 (14)	0.0712 (15)	−0.0154 (11)	0.0223 (12)	−0.0171 (11)
C23	0.0568 (11)	0.0632 (13)	0.0605 (13)	−0.0029 (10)	0.0187 (10)	−0.0101 (10)
C24	0.107 (2)	0.0684 (16)	0.0941 (19)	−0.0111 (15)	0.0384 (17)	−0.0337 (15)
S1	0.0548 (3)	0.0474 (3)	0.0663 (3)	0.0028 (2)	0.0013 (2)	−0.0028 (2)
S2	0.0431 (2)	0.0417 (2)	0.0565 (3)	0.00114 (18)	0.0131 (2)	−0.0028 (2)
N1	0.0474 (8)	0.0412 (8)	0.0643 (10)	−0.0011 (6)	0.0140 (7)	−0.0090 (7)
N2	0.0399 (7)	0.0448 (8)	0.0598 (10)	−0.0005 (6)	0.0127 (7)	−0.0110 (7)
O1	0.0781 (10)	0.0737 (11)	0.0641 (10)	−0.0086 (8)	−0.0031 (8)	−0.0132 (8)
O2	0.0706 (10)	0.0608 (10)	0.1129 (15)	0.0211 (8)	0.0075 (10)	0.0060 (10)
O3	0.0645 (9)	0.0673 (9)	0.0570 (8)	−0.0022 (7)	0.0239 (7)	−0.0007 (7)
O4	0.0549 (8)	0.0441 (8)	0.0862 (11)	0.0089 (6)	0.0111 (7)	−0.0004 (7)

### Geometric parameters (Å, °)

**Table:**

C1—C2	1.385 (3)	C16—C17	1.376 (3)
C1—C6	1.385 (2)	C16—H16	0.9300
C1—H1	0.9300	C17—C18	1.379 (3)
C2—C3	1.378 (3)	C17—H17	0.9300
C2—C14	1.510 (3)	C18—C19	1.360 (4)
C3—C4	1.380 (3)	C18—H18	0.9300
C3—H3	0.9300	C19—C20	1.374 (3)
C4—C5	1.377 (3)	C19—H19	0.9300
C4—H4	0.9300	C20—H20	0.9300
C5—C6	1.382 (3)	C21—C22B	1.473 (7)
C5—H5	0.9300	C21—N1	1.484 (3)
C6—C7	1.505 (3)	C21—C22A	1.509 (6)
C7—N1	1.473 (2)	C21—H21A	0.9700
C7—H7A	0.9700	C21—H21B	0.9700
C7—H7B	0.9700	C22A—H22A	0.9600
C8—C9	1.377 (3)	C22A—H22B	0.9600
C8—C13	1.384 (3)	C22A—H22C	0.9600
C8—S1	1.759 (2)	C22B—H22D	0.9600
C9—C10	1.385 (4)	C22B—H22E	0.9600
C9—H9	0.9300	C22B—H22F	0.9600
C10—C11	1.371 (4)	C23—C24	1.482 (3)
C10—H10	0.9300	C23—N2	1.485 (3)
C11—C12	1.355 (4)	C23—H23A	0.9700
C11—H11	0.9300	C23—H23B	0.9700
C12—C13	1.370 (4)	C24—H24A	0.9600
C12—H12	0.9300	C24—H24B	0.9600
C13—H13	0.9300	C24—H24C	0.9600
C14—N2	1.469 (2)	S1—O1	1.4235 (16)
C14—H14A	0.9700	S1—O2	1.4276 (16)
C14—H14B	0.9700	S1—N1	1.6220 (18)
C15—C16	1.381 (3)	S2—O4	1.4239 (14)
C15—C20	1.385 (3)	S2—O3	1.4274 (15)
C15—S2	1.7543 (18)	S2—N2	1.6224 (16)
			
C2—C1—C6	121.08 (17)	C17—C18—H18	119.8
C2—C1—H1	119.5	C18—C19—C20	120.4 (2)
C6—C1—H1	119.5	C18—C19—H19	119.8
C3—C2—C1	119.08 (17)	C20—C19—H19	119.8
C3—C2—C14	121.14 (18)	C19—C20—C15	119.5 (2)
C1—C2—C14	119.76 (17)	C19—C20—H20	120.3
C2—C3—C4	120.50 (19)	C15—C20—H20	120.3
C2—C3—H3	119.7	C22B—C21—N1	115.4 (3)
C4—C3—H3	119.7	C22B—C21—C22A	31.0 (3)
C5—C4—C3	119.87 (19)	N1—C21—C22A	110.3 (3)
C5—C4—H4	120.1	C22B—C21—H21A	128.4
C3—C4—H4	120.1	N1—C21—H21A	109.6
C4—C5—C6	120.67 (18)	C22A—C21—H21A	109.6
C4—C5—H5	119.7	C22B—C21—H21B	79.5
C6—C5—H5	119.7	N1—C21—H21B	109.6
C5—C6—C1	118.79 (18)	C22A—C21—H21B	109.6
C5—C6—C7	121.08 (17)	H21A—C21—H21B	108.1
C1—C6—C7	120.09 (17)	C21—C22A—H22A	109.5
N1—C7—C6	112.47 (15)	C21—C22A—H22B	109.5
N1—C7—H7A	109.1	H22A—C22A—H22B	109.5
C6—C7—H7A	109.1	C21—C22A—H22C	109.5
N1—C7—H7B	109.1	H22A—C22A—H22C	109.5
C6—C7—H7B	109.1	H22B—C22A—H22C	109.5
H7A—C7—H7B	107.8	C21—C22B—H22D	109.5
C9—C8—C13	120.4 (2)	C21—C22B—H22E	109.5
C9—C8—S1	119.95 (17)	H22D—C22B—H22E	109.5
C13—C8—S1	119.6 (2)	C21—C22B—H22F	109.5
C8—C9—C10	118.9 (2)	H22D—C22B—H22F	109.5
C8—C9—H9	120.5	H22E—C22B—H22F	109.5
C10—C9—H9	120.5	C24—C23—N2	112.85 (19)
C11—C10—C9	120.1 (3)	C24—C23—H23A	109.0
C11—C10—H10	120.0	N2—C23—H23A	109.0
C9—C10—H10	120.0	C24—C23—H23B	109.0
C12—C11—C10	120.7 (3)	N2—C23—H23B	109.0
C12—C11—H11	119.6	H23A—C23—H23B	107.8
C10—C11—H11	119.6	C23—C24—H24A	109.5
C11—C12—C13	120.2 (3)	C23—C24—H24B	109.5
C11—C12—H12	119.9	H24A—C24—H24B	109.5
C13—C12—H12	119.9	C23—C24—H24C	109.5
C12—C13—C8	119.7 (3)	H24A—C24—H24C	109.5
C12—C13—H13	120.2	H24B—C24—H24C	109.5
C8—C13—H13	120.2	O1—S1—O2	119.23 (11)
N2—C14—C2	111.00 (15)	O1—S1—N1	107.17 (10)
N2—C14—H14A	109.4	O2—S1—N1	107.05 (10)
C2—C14—H14A	109.4	O1—S1—C8	107.96 (11)
N2—C14—H14B	109.4	O2—S1—C8	107.99 (11)
C2—C14—H14B	109.4	N1—S1—C8	106.83 (9)
H14A—C14—H14B	108.0	O4—S2—O3	119.85 (10)
C16—C15—C20	120.34 (18)	O4—S2—N2	106.51 (9)
C16—C15—S2	119.65 (14)	O3—S2—N2	106.90 (9)
C20—C15—S2	119.98 (14)	O4—S2—C15	108.02 (8)
C17—C16—C15	119.26 (19)	O3—S2—C15	107.73 (9)
C17—C16—H16	120.4	N2—S2—C15	107.22 (8)
C15—C16—H16	120.4	C7—N1—C21	115.15 (17)
C16—C17—C18	120.1 (2)	C7—N1—S1	116.09 (13)
C16—C17—H17	119.9	C21—N1—S1	116.36 (14)
C18—C17—H17	119.9	C14—N2—C23	115.60 (17)
C19—C18—C17	120.4 (2)	C14—N2—S2	117.71 (13)
C19—C18—H18	119.8	C23—N2—S2	117.68 (13)
			
C6—C1—C2—C3	0.1 (3)	C9—C8—S1—O2	−167.52 (19)
C6—C1—C2—C14	−178.75 (16)	C13—C8—S1—O2	15.1 (2)
C1—C2—C3—C4	0.2 (3)	C9—C8—S1—N1	77.6 (2)
C14—C2—C3—C4	179.06 (18)	C13—C8—S1—N1	−99.8 (2)
C2—C3—C4—C5	−0.2 (3)	C16—C15—S2—O4	162.80 (16)
C3—C4—C5—C6	−0.1 (3)	C20—C15—S2—O4	−19.20 (19)
C4—C5—C6—C1	0.5 (3)	C16—C15—S2—O3	31.99 (18)
C4—C5—C6—C7	−177.20 (18)	C20—C15—S2—O3	−150.01 (16)
C2—C1—C6—C5	−0.5 (3)	C16—C15—S2—N2	−82.76 (17)
C2—C1—C6—C7	177.22 (16)	C20—C15—S2—N2	95.24 (17)
C5—C6—C7—N1	−112.7 (2)	C6—C7—N1—C21	71.0 (2)
C1—C6—C7—N1	69.7 (2)	C6—C7—N1—S1	−148.14 (14)
C13—C8—C9—C10	1.8 (4)	C22B—C21—N1—C7	−98.2 (6)
S1—C8—C9—C10	−175.61 (19)	C22A—C21—N1—C7	−131.5 (4)
C8—C9—C10—C11	−1.3 (4)	C22B—C21—N1—S1	121.0 (6)
C9—C10—C11—C12	−0.2 (5)	C22A—C21—N1—S1	87.7 (4)
C10—C11—C12—C13	1.3 (5)	O1—S1—N1—C7	48.65 (16)
C11—C12—C13—C8	−0.8 (5)	O2—S1—N1—C7	177.65 (14)
C9—C8—C13—C12	−0.7 (4)	C8—S1—N1—C7	−66.87 (15)
S1—C8—C13—C12	176.7 (2)	O1—S1—N1—C21	−170.94 (15)
C3—C2—C14—N2	120.3 (2)	O2—S1—N1—C21	−41.94 (17)
C1—C2—C14—N2	−60.8 (2)	C8—S1—N1—C21	73.54 (16)
C20—C15—C16—C17	0.4 (3)	C2—C14—N2—C23	−69.5 (2)
S2—C15—C16—C17	178.43 (17)	C2—C14—N2—S2	144.01 (14)
C15—C16—C17—C18	−0.4 (3)	C24—C23—N2—C14	114.9 (2)
C16—C17—C18—C19	0.6 (4)	C24—C23—N2—S2	−98.7 (2)
C17—C18—C19—C20	−0.8 (4)	O4—S2—N2—C14	−173.57 (14)
C18—C19—C20—C15	0.8 (3)	O3—S2—N2—C14	−44.33 (16)
C16—C15—C20—C19	−0.6 (3)	C15—S2—N2—C14	70.98 (15)
S2—C15—C20—C19	−178.58 (17)	O4—S2—N2—C23	40.68 (17)
C9—C8—S1—O1	−37.4 (2)	O3—S2—N2—C23	169.93 (15)
C13—C8—S1—O1	145.2 (2)	C15—S2—N2—C23	−74.77 (16)

### Hydrogen-bond geometry (Å, °)

**Table:**

*D*—H···*A*	*D*—H	H···*A*	*D*···*A*	*D*—H···*A*
C17—H17···O1^i^	0.93	2.57	3.409 (3)	151

Symmetry codes: (i) −*x*+1, −*y*, −*z*+1.
